# Supplementary material for: Patterns of Intron Gain and Loss in Fungi
Source: PLoS Biol. 2004 Nov 30;2(12):e422. doi: 10.1371/journal.pbio.0020422 (PMC532390; doi:10.1371/journal.pbio.0020422)
Supplement: Table S1 — Also available at http://genes.mit.edu/NielsenEtAl/. (4.3 MB ZIP). [file pbio.0020422.st001.zip › NielsenEtAl/html/1373.html]

AN0697.1.NCU04427.1.MG04906.1.FG04946.1


```
 CLUSTAL W (1.82) Multiple Sequence Alignments - Introns Inserted


Sequence 1: MG04906.1	867 aa
Sequence 2: FG04946.1	886 aa
Sequence 3: NCU04427.1	880 aa
Sequence 4: AN0697.1	837 aa
Alignment Length: 961 aa
Number Identitical Residues: 169 aa
Alignment Score (without introns) 11225


MG04906.1 	MEDLSRSEYPAML0AHLTPNQASQTLNERVKRILRTNTEIADWLQ0ERRKVEEQYIAGLG
NCU04427.1	MDDLSRAEYPAML0ASLQPNQAVQIFSDRVKRIAKVNQEIADWLQ0ERRRVEEQYVAGLR
FG04946.1 	MEDMARAEYPAML0AALQPGQAVQTLTERMKRMTRVNTEVADWLQ0ERRRVEDQYVQGLK
AN0697.1  	-MDLSRQEYPALL0ASLQPTQAATVLADRIRVINKVNADIADWLQ0ERRRIEEAYAQGLR
          	  *::* ****:* * * * **   : :*:: : :.* ::***** ***::*: *  ** 

MG04906.1 	KLALFKVPNANSELG2VFQPPWDRV~LHSIESIAASHQLYVSRIEKDVEHSLRGFQSR-P
NCU04427.1	KLLLFKVPNAASELG2VFQAPWDKI~LSSTDGIAASHQLYAQRIDKDVEQPLRSFQSR-K
FG04946.1 	KLTQFKVPNGQSELG2VFQAPWNRI~IDSTESIAHAHHQLADRIEKDIEHPLRNFSNR-K
AN0697.1  	KLANRPQLDNAAALG2IFQIPWQRI0K----------------IDEDVERPLREYNTKNK
          	**      :  : ** :** **:::                  *::*:*:.** :..:. 

MG04906.1 	EVAGMQTFVTNMQNMAKELDDSQTAADKANKKGG----KKAEAANTRLETATQQWESQAP
NCU04427.1	EMQNINTINANLQTMARELDDAAEKSEKLSKKGGKARSQKVDAAASRLEAASQQWESQAP
FG04946.1 	DFQNMNTMSNNLSTMAKDLDDSQNAVDKLTKKGGRANTSKVESATSKLESATGQWESQAP
AN0697.1  	ELASMPGVQNDLTALAKSVESSQKKVEKAKEKGPKG-ADKLAAAVSAAHDMSQQWESRAP
          	:. .:  .  ::  :*:.::.:    :* .:**  . :.*  :* :  .  : ****:**

MG04906.1 	FIYESLQALDEQRTNHLRDLLTQLQTHEVDQASRTQASAEEALNMTLEVDTAQEIQSFAS
NCU04427.1	FIFETLQALDEQRTNHLRDVLTQLQTHEVDQATRTQAAAEEVLNTMLEVNTGLEIHNFVS
FG04946.1 	FIFESLQALDESRVNNLRDLLTQYQTHESDSAQRVQENAMETLALMLEINTEKEIESFVH
AN0697.1  	FAFEQLQAADEGRLNHLRDVLTQLETHESDQIERCRQAAENCLNVLLNVETADEIKTFAA
          	* :* *** ** * *:***:*** :*** *.  * :  * : *   *:::*  **..*. 

MG04906.1 	RTVAGKPKQERR--AQMSRQTSAVASTTRPPSSAG--GLSGHQDDATN-------SSEHS
NCU04427.1	KVTAGRTKFERRTTTARQSSSAGGGSPPVGPPPGT----PLHDDDGMS---------QHS
FG04946.1 	RATAGKAKLPTRNSTRQSSFAGTTPSTPVPPSTADSTNLPAQSAHTASPAPVTAPTSSHA
AN0697.1  	KVNGGRPVVPLRQQTTSPTPVASPPPGPEPPLPPP----PRAQDDAAS-------HRSTS
          	:. .*:.    *  :      .   . .  * .      .  . .  .         . :

MG04906.1 	GGRLEPPTE1SK~LRS-~----RLGTMLG-RRRQSIHG-FGQLSPAKNAGPFSRGLSSAH
NCU04427.1	G--FRDQSD1SK~LRS-~----RIGTMLG-RRRQSIHGGFGQLGPPKALGAFTRGLGSSH
FG04946.1 	DDDMSETSM~PQ~EKPG1GKLRRLGTMFGGRRRQSMHAGFGQLSPGKLGPSFGR-LGTSH
AN0697.1  	SGRARPPPP~PV1PEP-~----RHGTPFGGLRRLGTVMNRRKSMALPSGPSSDKKYRSPF
          	..     .  .   ..      * ** :*. ** .   .  :  .     .  :   :..

MG04906.1 	GPR---MSPRASMNNLSESG--RLSSLAESPDSPRDGKAPETNGKTSHEGSTNGLKSKDE
NCU04427.1	GQT---LSPGGSSHNLAESHPHRLSSMAERPSTS-DERVKRDNINDFLHGGTNGFNSGDG
FG04946.1 	SQTDRGPSPRASSNNLQEHQ--RLSSLAETPDMPRVPDGENDTPKPDAHENANGTSHNEN
AN0697.1  	SSFK-----RGDSHNMQIPE--------DPPERPDTAITSQDSYNETARQPSESYEREDR
          	.  .      .. :*:            : *. .      . . :   .  ::. .  : 

MG04906.1 	GSAAAHAGESGSTSKPALTNGEPTADDLFGPPPAASQADNETGNNV~GGKDADGFTVPPE
NCU04427.1	QAESSQPRPTSNNLTNGTAENIFDAAPS-APPASTQQSQP---HEE~PAKDAEGYTIPQY
FG04946.1 	LLDSPIPQSAGGTVNGNHEIDLSNVQPPPGPPPSHKQDQPSAPQNA~T-KDSDGFNVPPP
AN0697.1  	ARFSPSPPPQSTPDTNGFASQ-----------GPAATAGLTGVTNE0PRVDAEGYSERPQ
          	   :. .   .   .                  .          :     *::*:.    

MG04906.1 	MNDPISQAQKEA---AGD~DHDQFFKLNIQKEPVAEEDADAQKAAISNVTNTLS-~MSMP
NCU04427.1	SHDPIAEAQKEA---AAE~EPDHLFKLNIQNEPIAEEDQDAKQAALSNVAHTLTA~MGLP
FG04946.1 	MNDPISEAQREAKQESGE~DPDQLLKVNIQQTPVEDEDPDAKMAALSSVANSLK-~MGPA
AN0697.1  	TIDEITLAQREA----AG2HEESGLNLTIRDQPIP-EDENQAQQAMNDMASQLRL0QAQQ
          	  * *: **:**    .  . :  :::.*:. *:  ** :    *:..::  *    .  

MG04906.1 	S---RKSGTIRGRRDVRNTIYMPPPAMPDAGSQLGTIPASPASPSLPTSVSKPSAVATLA
NCU04427.1	N---RKAGTVRGRRDVRHTMYIP--AMPVPDHPENPFPSSPSLPATASSIIRSTPPITLG
FG04946.1 	T---RRSGTVRGRRDVRHTMYVPPPNLPESGTDS-PISGLAASPILAPSTSKGSALAALA
AN0697.1  	SGIRRNAGTIRGRRDVRNTVFIPSTPPPVHPVPNQAVAQDVSEPTSPTSPGRHVASLSNA
          	..  *.:**:*******:*:::*..  *       ...   : *  ..*  :  .  : .

MG04906.1 	SEAS-VAAMSDTQSVRSSNSLNSL------AHAKHPELT----APGLSSSIIETVSAHFE
NCU04427.1	SEPSRASHASDNQSIRSGTSYGAGSAYGGIAHLKHQDIHGPDFGPGLHSSIIETVSALFQ
FG04946.1 	SESS-IAGTSDTQSVRSGNSLGGG------NHPQHAHMT----APGLNSSIIESVSAVFE
AN0697.1  	TDEH---ALSDTTSIRSSHTLHSIS-----GPVAHPELH----DSGLNASIIETVNAWFS
          	::       **. *:**. :  . :         * .:      .** :****:*.* *.

MG04906.1 	GGNLKTVKVNGEIAFAYN---------~--------------AGPD--FIPKG1------
NCU04427.1	DGEAKSVKVTGEIALSYKSDPHNLQTD1NQIIRLNNFARLESIGPNRLFVTSD~AT----
FG04946.1 	DGVIKSASITGEVAFVNNPSDSGDAKT1YETIRINNFAALERIGPNRIFVQNS~SPD---
AN0697.1  	EGAVTKSFVVGELALAFNAAPDVLSDK~IR-VRLNNFPVLEKVAANPNFIREV~QDQSED
          	 *  ..  : **:*:  :.               .. .  .  ..:  *: .    .:..

MG04906.1 	YSDEFTLDTSHMS-KTAT-AFTYRLHCEPEDPSTLEPLCPLIVKPAWKPTQDHKIGLLLQ
NCU04427.1	SPDEFTINTSHLT-TISTPAFTYRVHAEND-TALVSSHCPISINPVWKP-QGDKLGLLLQ
FG04946.1 	HHDQFNIDVSHLT-KNAT-AFSYRVFEESE-ASVLAEHAPLLLKPAWKP-QGDKLGLLLQ
AN0697.1  	KRGEYDVSLASITRSAPTVAFKYQVHINPE---DLSTFCPVIFRPAWNL-EEFQASVIIF
          	  .:: :. : :: . .* **.*::. : :    :   .*: ..*.*:  :  : .::: 

MG04906.1 	YALNPAIFPSIDGAASSPTSETSSSLPQTITLRNLVFVATYEG-----------RASAAQ
NCU04427.1	YRLNP-----------------SVTLQRPIVLHNVTFVATYEGA----------RASGVQ
FG04946.1 	YQLNP-----------------ASKIAAPVSLHNVVIVAKYEG-----------KSSGAQ
AN0697.1  	YSLNP--------------SFASAASKDSITLTNLVLTVNLDISPQEDREVAHATNAVMY
          	* ***              : ::     .: * *:.:... : :.... . : :  :   

MG04906.1 	TKPAGTHLRDKHLVYWRLGDVTLTCGSEIEWSKIVCRIVGADGADIQPGHIEARWEYTPQ
NCU04427.1	TKPSGTHLKEKHLVYWRIPELTLTD----DWAKIICRVIGEQNGEPQPGHIEVRWEYTAP
FG04946.1 	TKPVGIHLKDKHLVYWRIGDLTLTN----DPQKIVCRIIGSEGTEPKPGHIEARWELTAS
AN0697.1  	PNTGAVFRRKQSAVIWKIPEFEVNS----KSDGKLLVRFSTANSWPQKGKVEAKFEVRTP
          	.:. . . :.:  * *:: :. :.     .    :   ..  .   : *::*.::*  . 

MG04906.1 	ASSSAPR-ISISRLLEDTKGKGKAVVEEDGTLSAGDPFSDDNP---ISPRNDDRKWAEVP
NCU04427.1	PAGEVGSGISVSRLVEG-KGKEKAEEQSE----EEDPFADDKGPISPALGKDGRTWAEVP
FG04946.1 	GDQPVGSGISISRLDEG-KGKGKEISDDD-------PFADDS-------ASTEQKWVDVP
AN0697.1  	DAGSRLGISSASRPAGS------------------DPFADED--------NAQAQWSHVP
          	         * **   .                  .**:*:.        .    * .**

MG04906.1 	LVRKAVSGK~YEAK~
NCU04427.1	LSRKLIGGK~YEAK~
FG04946.1 	VSRKLVSGK~YEGR~
AN0697.1  	TTRKLAVGK2VAYM1
          	  **   **
```
